# Transcriptomic characterization and innovative molecular classification of clear cell renal cell carcinoma in the Chinese population

**DOI:** 10.1186/s12935-020-01552-w

**Published:** 2020-09-22

**Authors:** Qiang Zhao, Jia Xue, Baoan Hong, Wubin Qian, Tiezhu Liu, Bin Fan, Jie Cai, Yongpeng Ji, Jia Liu, Yong Yang, Qixiang Li, Sheng Guo, Ning Zhang

**Affiliations:** 1 Key laboratory of Carcinogenesis and Translational Research (Ministry of Education/Beijing), Department of Urology, Peking University Cancer Hospital & Institute, 52 Fucheng Road, Haidian District, Beijing, 100142 People’s Republic of China; 2 Systems Biology, Crown Bioscience Inc., No. 218 Xinghu Street, Suzhou Industrial Park, Suzhou, 215028 People’s Republic of China; 3 Department of Urology, Daqing Oilfield General Hospital, Heilongjiang, 163316 People’s Republic of China; 4 Crown Bioscience Inc., No.21 Huoju Street Changping District, Beijing, 102200 People’s Republic of China; 5 Crown Bioscience Inc., 3375 Scott Blvd, Suite 108, Santa Clara, CA 95054 USA; 6 State Key Laboratory of Natural and Biomimetic Drugs, Peking University, Beijing, 100191 People’s Republic of China

**Keywords:** Transcriptomic characterization, Molecular classification, Clear cell renal cell carcinoma, TCGA, Immuno-phenotyping

## Abstract

**Background:**

Large-scale initiatives like The Cancer Genome Atlas (TCGA) performed genomics studies on predominantly Caucasian kidney cancer. In this study, we aimed to investigate genomics of Chinese clear cell renal cell carcinoma (ccRCC).

**Methods:**

We performed whole-transcriptomic sequencing on 55 tumor tissues and 11 matched normal tissues from Chinese ccRCC patients. We systematically analyzed the data from our cohort and comprehensively compared with the TCGA ccRCC cohort.

**Results:**

It found that PBRM1 mutates with a frequency of 11% in our cohort, much lower than that in TCGA Caucasians (33%). Besides, 31 gene fusions including 5 recurrent ones, that associated with apoptosis, tumor suppression and metastasis were identified. We classified our cohort into three classes by gene expression. Class 1 shows significantly elevated gene expression in the VEGF pathway, while Class 3 has comparably suppressed expression of this pathway. Class 2 is characterized by increased expression of extracellular matrix organization genes and is associated with high-grade tumors. Applying the classification to TCGA ccRCC patients revealed better distinction of tumor prognosis than reported classifications. Class 2 shows worst survival and Class 3 is a rare subtype ccRCC in the TCGA cohort. Furthermore, computational analysis on the immune microenvironment of ccRCC identified immune-active and tolerant tumors with significant increased macrophages and depleted CD4 positive T-cells, thus some patients may benefit from immunotherapies.

**Conclusion:**

In summary, results presented in this study shed light into distinct genomic expression profiles in Chinese population, modified the stratification patterns by new molecular classification, and gave practical guidelines on clinical treatment of ccRCC patients.

## Background

Kidney cancer, or renal cell carcinoma (RCC), accounts for about 2–3% of tumor malignancy in adults, and is one of the most lethal urological cancers [1]. Clear cell renal cell carcinoma (ccRCC) is its most common subtype (75–85%). According to the data from the Chinese National Cancer Registry Center, over 67,000 new cases were diagnosed and 23,400 died in China in 2016, and these numbers are rapidly increasing each year. Due to the improvement of people’s health consciousness and the upgrades of surgical techniques, 5-year survival rates of organ-confined disease can reach 70–90% [2]. Patients with lymph node or systemic metastases suffer comparably worse with 5- and 10-year survival rates of 5–30% and 0–5%, respectively [3–5].

Besides clinical and histopathological features, RCC can be also characterized by underlying genomic variations and high immune infiltration [6]. Genomic characterizations of resource datasets on RCCs have been performed in the past few years, including clear cell renal cell carcinoma (ccRCC) [7–9], chromophobe renal cell carcinoma [10], papillary renal cell carcinoma [11] and renal medullary carcinoma [12]. One integrative taxonomy research has been performed to assemble three TCGA renal carcinoma subtypes [13].

Race and ethnicity cause inter-tumoral heterogeneity in cancers, ranging from disease incidence, morbidity, and mortality rates to treatment outcomes [14, 15]. Therefore, identification of population-specific molecular biomarkers is very important to this end. For example, Shi et al. utilized TCGA data to perform an integrative comparison between Caucasian and Asian Americans on gene expression patterns in breast cancers and found significant differences at gene and pathway levels [16]. But global genomic and transcriptomic similarities and dissimilarities between Asian and Caucasian for ccRCC remain largely unknown.

Cancers are increasingly recognized as collections of diverse not only genetic diseases, but also immune diseases. The heterogeneous tumor microenvironment (TME), including immune components, plays critical roles in tumor growth, progression and response to pharmaceutics, particularly immuno-oncology therapeutics. In recent years, immuno-phenotyping studies based on high-throughput assays on bulk tissue or single cell levels become more popular in oncology research [9, 17–20]. Recently, two studies provided an immune atlas of ccRCC as inflammatory subtype of tumors by genomic analysis on bulk tumor level and by mass spectrometry single-cell level, respectively [17, 20]. It would be interesting to understand the underlying mechanisms for universal tumor-immune interactions in ccRCC.

To this end, we performed whole-transcriptome sequencing (WTS) on a cohort of 66 samples derived from 55 tumor tissues from Chinese ccRCC (CccRCC) patients and 11 of their matched normal tissues. We compared genetic variations and gene expression of CccRCCs with other large cohorts from TCGA and identified novel genomic features for ccRCCs in Chinese patients. Additionally, we uncovered new immunological characteristics for ccRCC progression and repression.

## Materials and methods

### RNA isolation and WTS on CccRCC

Freshly and surgically removed tumors and non-cancerous matched tissues (normals) were obtained from 55 patients diagnosed as ccRCC (These patients were enrolled from August 26th, 2016 to July 24th, 2017.) and frozen for storage. Total RNAs were extracted by RNeasy kit from (Qiagen) and the purity and integrity of the RNA samples (RIN > 7 and 28S/18S > 1) were determined by Agilent Bioanalyzer prior to sequencing. polyA + mRNA sequencing was performed by certified service providers at paired-end 150 bp on Illumina HiSeq platform.

### External datasets retrieval and processing

Level-3 TCGA RNA-seq data on kidney renal clear cell carcinoma or KIRC (533 tumor and 72 normal samples) were downloaded from the TCGA data portal (February 2015 release, https://tcga-data.nci.nih.gov/tcga/). RNA-seq data generated by the Illumina HiSeq platform were used and processed by the RNAseqV2 pipeline, which used MapSplice [21] for read alignment and RSEM for quantification [22]. Clinical metadata of the three cancer types were obtained from the TCGA data portal (https://portal.gdc.cancer.gov, November 2017) and converted to tab-delimited text tables.

### Bioinformatics analysis on RNA-seq data

#### Gene mutation analysis

Raw RNA-seq reads passed the FastQC tool (https://www.bioinformatics.babraham.ac.uk/projects/fastqc/) default filters were aligned to the human genome assembly hg19. Somatic mutations on expressed genes were detected from aligned data for 55 tumor samples by the STAR mapping software [23] and the GATK variant discovery toolkit [24]. Several filtering steps were performed to exclude low-quality and germline mutations: (1) low-quality candidate events (i.e., the mutation events not tagged as PASS by GATK or with alternative allele depth smaller than 5, and indel mutations in poly-N region or with alternative allele rate less than 20%) were removed; (2) The mutation events that were observed in the 1000 Genome Project [25] and the 6500 Exome Project [26] with greater than 0.5% frequency were excluded; (3) the putative events should be within exonic regions and be protein-changing; (4) only mutations associated with cancer related genes (572 cancer consensus genes from COSMIC database (https://cancer.sanger.ac.uk/cosmic) were retained [27]. The 11 matched normal samples were used as reference to further check the reliability of the detected somatic mutation. The remained putative mutations were used to infer driver mutations via a web tool—the Cancer Genome Interpreter (https://www.cancergenomeinterpreter.org/). To compare the overall mutation frequencies of driver mutation genes in the Chinese and Caucasian populations, Fisher’s exact test was performed on each driver genes with 2 × 2 matrix of the sample counts with or without respective mutation in two datasets.

#### Gene fusion detection

Gene fusion information was detected by SOAPfuse [28] and Fusioncatcher [29] using mapped BAM files. Fusion events that are recurrent in more than one tumor sample were visualized as a circos plot [30].

#### Gene expression data preprocessing and basic analysis

Gene expression was estimated by the MMSEQ software [31] and raw gene counts were normalized by the RSEM software [22]. For the cross-dataset comparison of kidney cancer cohort for TCGA patients and CccRCC patients, “sva” package was utilized to estimate batch effect before pairwise Spearman correlation analysis on corresponding transcriptome [32]. “Rtsne” package was employed to ensure absence of batch effect between two datasets [33]. Differentially expressed (DE) genes and pathways between different clinical groups (Asian versus white, early pathologic stage versus late stage, lower histologic grade versus higher grade) were identified by using “Limma” and “GSVA” package with different cutoffs [34, 35]. Genes sets are derived from KEGG (https://www.genome.jp/kegg/), REACTOME (https://reactome.org/) and BIOCARTA (http://www.biocarta.com/) databases, which are integrated by MSigDB [36].

### RT-PCR and Sanger sequencing validation

The genomic DNA of tumor and blood samples were isolated using DNeasy Blood and Tissue Kit (Qiagen Cat#69506) following manufacturer’s instruction. PCR amplification of amplified the mutation sites of *PBRM1*, and Sanger sequencing was used to verify the status of mutation sites. The PBRM1 primer sequences are provided in Additional file 1.

### Molecular classification of ccRCC

The gene expression data matrix was z-score transformed and the maximum absolute deviation was calculated for each gene to select the top variable genes for clustering. The data were then transformed into a non-negative matrix and clustered using the “NMF” package [37]. First, we tested a series of gene numbers (1500, 2000, 3000, 4000, 5000, 6000 and 7000). Rank estimates were calculated using 50 iterations of ranks 2–8 with default settings. 3000 genes give best clustering with k = 3. DE genes across NMF-derived subtypes were identified by comparing each class with other two and Top 300 DE genes ranked by adjusted p-value among all comparisons were retained with the best recall of original NMF-based clustering using 3000 genes. Pathway enrichment analysis was performed on 300 DE genes using REACTOME 2016 pathway database via Enrichr webtool (http://amp.pharm.mssm.edu/Enrichr/) [38, 39]. Pairwise Spearman correlation (COR) and distance matrix (1-COR) based on DE genes were determined for CccRCC samples (n = 55) combined with KIRC samples from TCGA (n = 533) as a complete cohort. Hierarchical clustering of distance matrix was performed to identify predicted classes. The classified KIRC samples were used for survival analysis.

### Transcriptional immuno-phenotyping analysis

The mRNA expression of 66 immune markers proposed by TCGA Network [40] was plotted as a heatmap for all CccRCC samples (55 tumors and 11 normals) and TCGA KIRC samples separately, and hierarchical clustering was performed. Survival analysis of TCGA patients were then performed for immuno-stratified groups. The RSEM-normalized expression matrix of all genes for all CccRCC samples was utilized for deconvolution analysis by EPIC algorithm [41]. The signature scores of 6 human hematopoietic cell types were displayed as a stacked bar plot, where samples are ordered in the same immuno-stratified groups. Volcano plots showing log10 mean ratio and p-value between groups were drawn by comparing the inferred relative fractions from immune-active and tolerant tumors (n = 51) versus immune-inactive tumors and normal samples (n = 15).

## Results

### Patient clinical information

Transcriptome sequencing was performed on 55 tumor tissue samples from CccRCC patients, 11 of which with matched normal tissues. The clinical information, including age, gender, race, vital status, metastasis, pathologic stage and histologic grade, was summarized in comparison with the TCGA ccRCC cohort (Table 1). To note, our cohort is a slightly biased in early stages (Stage I: 50.1%; Stage II: 10.7%) and moderate grades (Grade 2: 43.0%; Grade 3: 38.6%) biased compared with the TCGA cohort and hitherto all patients are alive.

**Table 1:** Clinical data summary of studied ccRCC datasets

	CKC dataset	Percentage	TCGA dataset	Percentage
Sample (matched normal)	55 (11)		533 (72)	
Average of age (range)	58.3 (25–80)		60.63 (26–90)	
Gender				
Male	41	74.5	345	64.7
Female	14	25.5	188	35.3
Race				
White	0	0.	462	86.
Black	0	0.0	56	10.5
Asian	55	100.0	8	1.5
Not available	0	0.0	7	1.3
Vital status				
Alive	55	100.0	358	67.2
Dead	0	0.0	175	32.8
Metastasis				
Mets−	51	92.7	422	79.2
Mets+	4	7.3	109	20.5
Not available	0	0.0	2	0.4
Pathologic stage				
Stage I	41	74.5	267	50.1
Stage II	3	5.5	57	10.7
Stage III	11	20.0	123	23.1
Stage IV	0	0.0	83	15.6
Not available	0	0.0	3	0.6
Histologic grade				
Grade1	8	14.5	14	2.6
Grade2	29	52.7	229	43.0
Grade3	13	23.6	206	38.6
Grade4	4	7.3	76	14.3
Not available	1	1.8	3	0.6

### Driver mutations and gene fusions

**Fig. 1:**
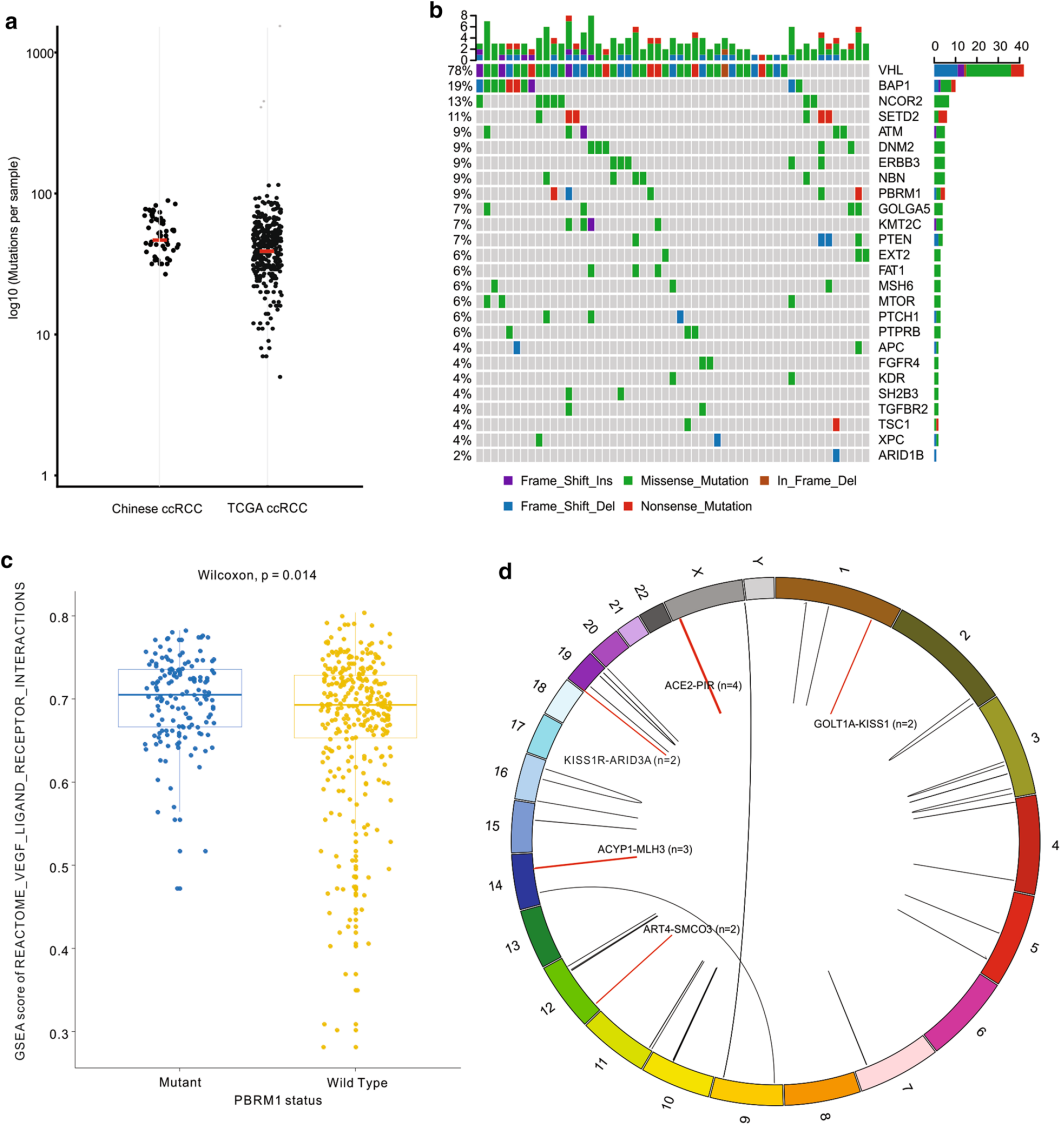
Driver mutations and Gene fusion markers. **a** Scatterplot of mutation load in clear cell renal cell carcinoma patients from Chinese (n = 55) and TCGA (n = 533) collection. Median mutation load of each dataset is marked in red. **b** Summary of Top 26 driver mutations detected in Chinese cohort. Colors indicate mutation types. **c** *PBRM1* mutation is associated with activation of *VEGF* signaling pathways in TCGA ccRCC collection. **d** Circos plot of detected fusion genes. Recurrent fusion events observed in more than one sample are highlighted in red

To investigate population-specific genomic features, important driver mutations and recurrent gene fusions were identified in CccRCC tumors (Fig. 1). The median number of estimated somatic mutations for ccRCC patients is 54 per sample, at a comparable level to the TCGA cohort (KIRC: 44) (Fig. 1a). Due to lack of paired normal controls and the limitation of WTS technology, the mutation frequencies for the vast majority of important ccRCC variant genes, such as *VHL*, *BAP1* and *SETD2*, were shown in higher levels than in the TCGA cohort, except for *PBRM1*, a second most significantly mutated gene (SMG) (33.7% in the Caucasian population) as previously described [8, 42], which was observed with only 14.5% mutation rate in Chinese patients (Additional file 2: Table S1a). RT-PCR and Sanger DNA sequencing confirmed that the true mutation rate of *PBRM1* is 10.9%. We further identified 191 putative driver mutations associated with 572 cancer consensus genes from the COSMIC database [27] (Additional file 2: Table S1b). The most frequently mutated driver genes are *VHL* (78%), *BAP1* (19%), *NCOR2* (13%) and *SETD2* (11%) in 53 samples with driver mutation (Fig. 1b). Fisher’s exact test was then performed on each driver gene to investigate the overall mutation frequency difference between the Chinese and Caucasian populations. Significant difference (*p*-value < 0.05) in mutation frequencies between two populations was observed in 11 out of 84 driver genes, of which 3 were previously proposed SMGs in ccRCC [42], including *VHL* (*p*-value = 8.6E−4), *PBRM1* (*p*-value = 8.4E−5) and *KDM5C* (*p*-value = 3.8E−2) (Table 2). Further mRNA expression analysis indicated that *PBRM1* mutation is associated with activation of the VEGF signaling pathways (Fig. 1c).

**Table 2:** The frequencies of driver mutation genes in CccRCC and TCGA datasets

Gene	Frequency in Chinese ccRCC (%)	Frequency in TCGA KIRC white (%)	p-value (Fisher’s exact test)
VHL	76.4	52.8	0.000862049
BAP1	18.2	9	0.053248208
NCOR2	12.7	1.4	0.000127079
SETD2	10.9	11	1
PBRM1	9.1	33.7	8.38E−05
ATM	9.1	3.5	0.065990135
ERBB3	9.1	1.9	0.011804502
NBN	9.1	0.4	0.000226619
DNM2	9.1	0.2	7.06E−05
PTEN	7.3	4.3	0.299167676
KMT2C	7.3	3.8	0.269293981
GOLGA5	7.3	1.1	0.010440507
MTOR	5.5	7.1	1
FAT1	5.5	3.5	0.449134307
PTCH1	5.5	2.1	0.131792795
MSH6	5.5	1.1	0.045880562
PTPRB	5.5	0.9	0.031041033
EXT2	5.5	0.4	0.01040418
APC	3.6	1.1	0.170756605
KDR	3.6	1.1	0.170756605
XPC	3.6	1.1	0.170756605
TSC1	3.6	0.9	0.13083787
FGFR4	3.6	0.7	0.093628013
SH2B3	3.6	0.4	0.06033848
TGFBR2	3.6	0.4	0.06033848
FANCE	1.8	4.3	0.711854112
PIK3CA	1.8	4	0.70899921
TP53	1.8	3.3	1
POLE	1.8	3.1	1
CHD4	1.8	2.6	1
SMARCA4	1.8	2.3	1
ATR	1.8	2.1	1
CDK12	1.8	2.1	1
TET2	1.8	2.1	1
CHEK2	1.8	1.9	1
EGFR	1.8	1.9	1
BLM	1.8	1.6	0.604409035
FLT4	1.8	1.6	0.604409035
GNAS	1.8	1.6	0.604409035
MYH11	1.8	1.6	0.604409035
TRRAP	1.8	1.6	0.604409035
NCOR1	1.8	1.4	0.55540565
PIK3CB	1.8	1.4	0.55540565
FBXW7	1.8	1.1	0.500455786
GATA2	1.8	1.1	0.500455786
KAT6B	1.8	1.1	0.500455786
MLLT4	1.8	1.1	0.500455786
TCF12	1.8	1.1	0.500455786
ERBB2	1.8	0.9	0.4388528
ARID1B	1.8	0.9	0.4388528
ASXL1	1.8	0.9	0.4388528
CIC	1.8	0.9	0.4388528
JAK3	1.8	0.9	0.4388528
MECOM	1.8	0.9	0.4388528
NUP98	1.8	0.9	0.4388528
PER1	1.8	0.9	0.4388528
BARD1	1.8	0.7	0.369807843
DDR2	1.8	0.7	0.369807843
MET	1.8	0.7	0.369807843
NDRG1	1.8	0.4	0.292440502
FANCG	1.8	0.4	0.292440502
PLCG1	1.8	0.4	0.292440502
PMS2	1.8	0.4	0.292440502
PRF1	1.8	0.2	0.205768403
CEBPA	1.8	0.2	0.205768403
MAP2K1	1.8	0.2	0.205768403
MYH9	1.8	0.2	0.205768403
PRDM1	1.8	0.2	0.205768403
TCF7L2	1.8	0.2	0.205768403
FH	1.8	0	0.108695652
CDKN1B	1.8	0	0.108695652
KLF6	1.8	0	0.108695652
PPM1D	1.8	0	0.108695652
TRAF7	1.8	0	0.108695652
KDM5C	0.0	6.9	0.037678184
ARID1A	0.0	5.5	0.095406442
MALAT1	0.0	2.1	0.606792113
FGFR3	0.0	1.4	1
ABL2	0.0	1.1	1
BCL6	0.0	1.1	1
CDK4	0.0	0.9	1
CDH1	0.0	0.7	1
PALB2	0.0	0.7	1
FANCF	0.0	0.2	1

Fisher’s Exact test was performed for each gene by comparing the sample counts with mutant and wild type in two datasets

Thirty-one fusion events were detected in the CccRCC patients, five of which were observed in more than one sample (Fig. 1d). Functional analysis on the downstream related genes, e.g. *PIR* for the *ACE2*-*PIR* fusion, revealed their association with apoptosis, cancer suppression and metastasis, etc. (Additional file 3: Table S2). However, none of the fusion events was observed in TCGA patients [8].

### Expression across populations and transcriptional variations during tumor development

**Fig. 2:**
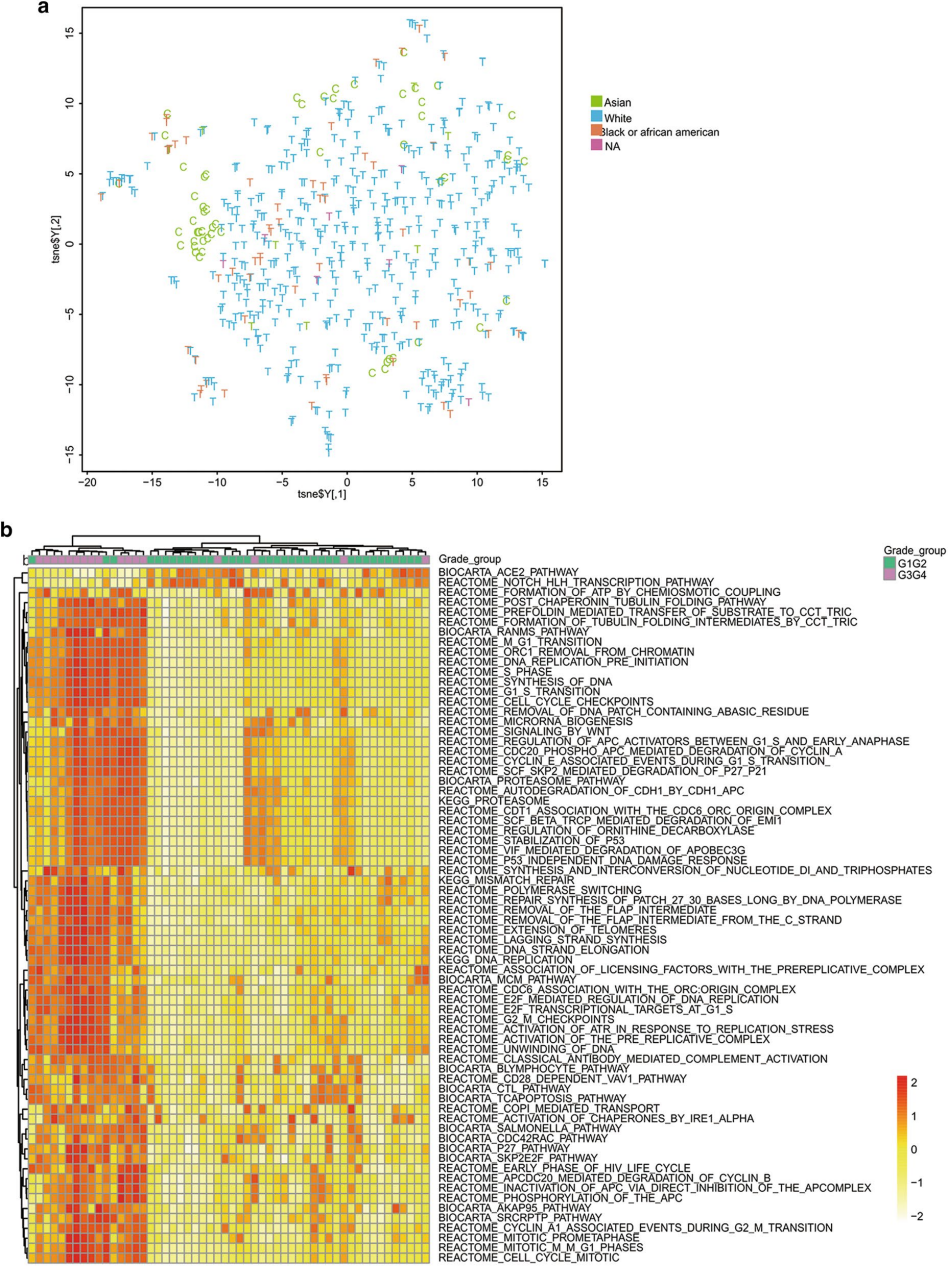
Global overview of the transcriptomics of ccRCC patients. **a** The t-Distributed Stochastic Neighbor Embedding (t-SNE) plot of global mRNA expression for Chinese (C, n = 55) and TCGA’s (T, n = 533) ccRCC patients. Samples are colored by race. **b** Heatmap for gene set variation analysis (GSVA) on early (T1T2) and late (T3T4) clinical stages. Cutoffs used for GSVA were:|Fold Change| > 1.3 and Bonferroni & Hochberg adjusted p-value < 0.05

The gene expression profiles from both Chinese (n = 55) and TCGA (n = 533) ccRCC cohorts were integrated and compared for population specific variations. The t-Distributed Stochastic Neighbor Embedding (t-SNE) plot of the expression profiles from both datasets demonstrated a largely uniform distribution of Asian, white and black patients, suggesting low influence of race on transcriptomic level inter-tumoral heterogeneity (Fig. 2a).

To examine the transcriptomic variation between patients with different tumor stages/grades, we used gene set variation analysis (GSVA) on the 55 tumor samples from Chinese ccRCC patients on 833 well-curated biological pathways. We observed a strong clustering of tumor samples by tumor grade, showing by 71 differentially expressed gene sets identified between grade groups (|Fold change| > 1.3, p-value < 0.05, Fig. 2b). A considerable number of pathways, especially pathways related to cell cycle, were significantly up-regulated in severe grade levels (Grade 3 and 4) than in mild level (Grade 1 and 2) (|Fold change| > 1.3, p-value < 0.05). Moreover, 91 differentially expressed gene sets are identified for stage groups (|Fold change| > 1.3, p-value < 0.05). The association between expression and tumor stages seems weak from clustering of pathways and genes (Additional file 4: Figure S1, Additional file 5: Figure S2).

### ccRCC classification by mRNA expression

There have been several molecular classification schemes of ccRCC [7, 8, 13]. Most recently, ccRCC was classified into 3 subtypes using multilevel genomics data [13]. Such classifications, however, are sensitive to the number of genes used for statistical computation. Therefore, we used a robust iterative method to classify the Chinese ccRCC patients (see “Materials and methods”). The expression signatures obtained therefrom, if were more clinical-relevant than existing ones, should classify the TCGA ccRCC patients into groups with more distinct survival patterns, especially that we observed no pronounced population-specific transcriptomic profiling between the two patient cohorts.

**Fig. 3:**
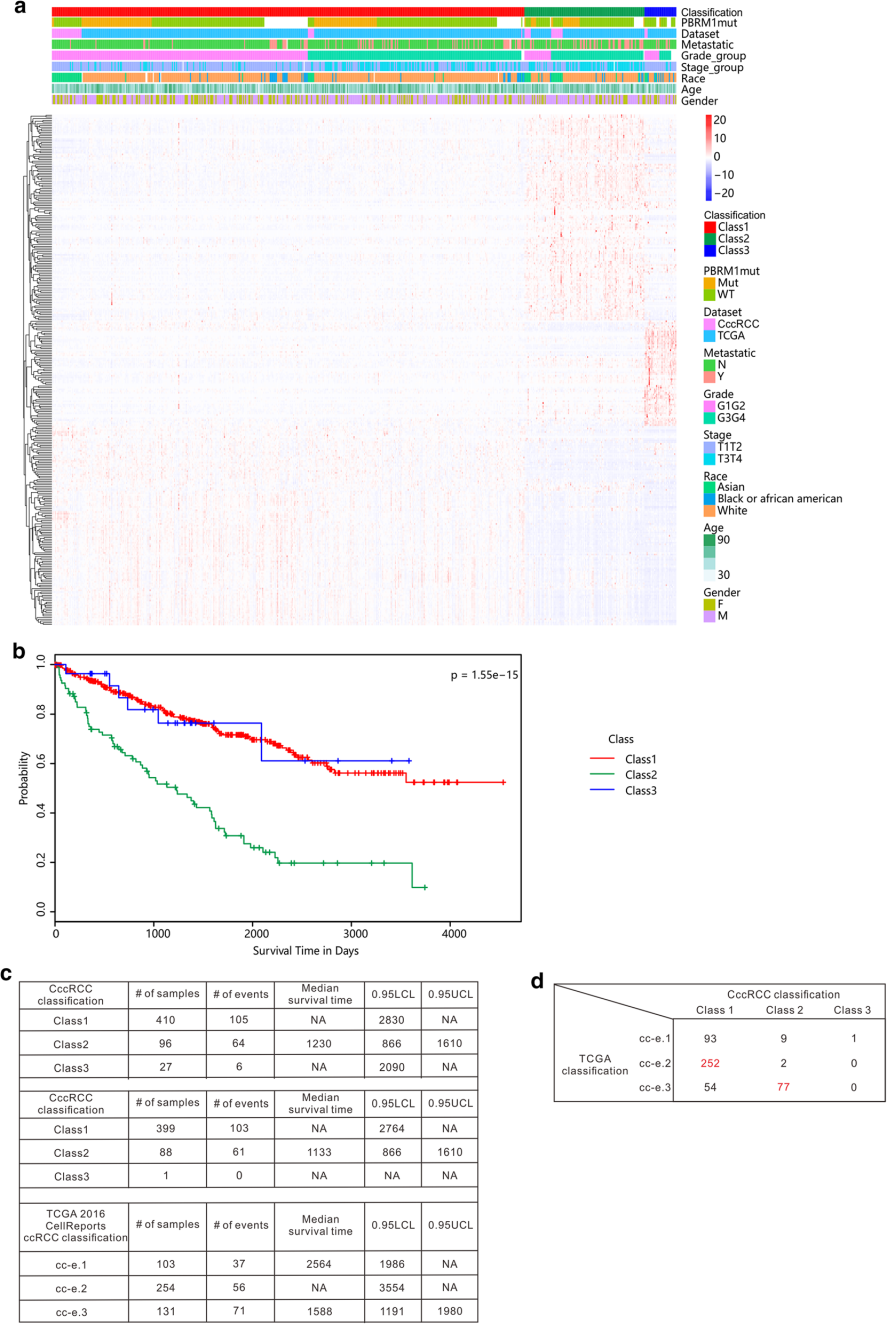
Clear cell renal cell carcinoma classification**. a** Chinese and TCGA ccRCC patients (n = 588) are classified into three subtypes based on NMF-clustering derived 300 genes. **b** Survival analysis on three identified classes of TCGA ccRCC patients revealed significant difference. The log rank test *p*-value across groups is 1.55E−15. **c** Comparison of CccRCC-defined ccRCC classification (n = 533 and n = 488) with TCGA’s classification (n = 488) by stratification of survival curves. **d** Overlap of samples classified in CccRCC and TCGA’s classification. The largest intersection for Class 1-3 is highlighted in red

Using our new method, the 55 CccRCC patients were clustered into 3 classes based on 3000 variable genes by unsupervised learning using the non-negative matrix factorization (NMF) algorithm (Additional file 6: Figure S3). Through iterations, top 300 differentially expressed genes were chosen as signature genes from the NMF-derived classes that, gave 94.5% agreement with that done by the original 3000-genes (Fig. 3a, Additional file 7: Figure S4). The most significantly enriched pathways for the 300 signature genes are extracellular matrix organization, hemostasis, and VEGF associated pathways, etc. (Additional file 8: Table S3).

The 300 signature genes were then used to classify TCGA ccRCC patients into 3 classes. Survival analysis of the three classes demonstrated significant difference in survival (log-rank test *p*-value = 1.55E−15), more so than that by the TCGA defined 3 subtypes, namely cc-e.1, cc-e.2 and cc-e.3 (log-rank test *p*-value = 8.94E−8) [13]. Gene set enrichment analysis revealed that Class 1 tumors are characterized by significantly elevated VEGF pathway genes whereas those in Class 3 are comparably depleted (Kruskal–Wallis test *p*-value = 4.9E−16, data not shown). Class 2 tumors, which possess increased expression of extracellular matrix organization genes (Kruskal–Wallis test *p*-value = 1E−15, data not shown), were strongly associated with higher grade of tumors, and thus resulted in the worst overall survival (Fig. 3b). Comparison of median survival time for each patient group from our and TCGA classifications demonstrated better indication of survival groups in our classification (Fig. 3c). The median survival time of our Class 2 patients was only 1230 days, much shorter than the other two groups. In the TCGA classification, the cc-e.3 subtype had the worst survival, but its median survival was a much longer 1588 days. Cross comparison of the two classification schemes reveals that the majority (77 out of 88) of our Class 2 patients were in the TCGA cc-e.3 subtype, which however had an additional 54 other patients. Further, Class 1 patients have overlap with all three TCGA subtypes and dominant in cc-e.2 whereas Class 3 seems to be a rare novel subtype that has not been discovered in previous studies.

### Immuno-phenotyping

**Fig. 4:**
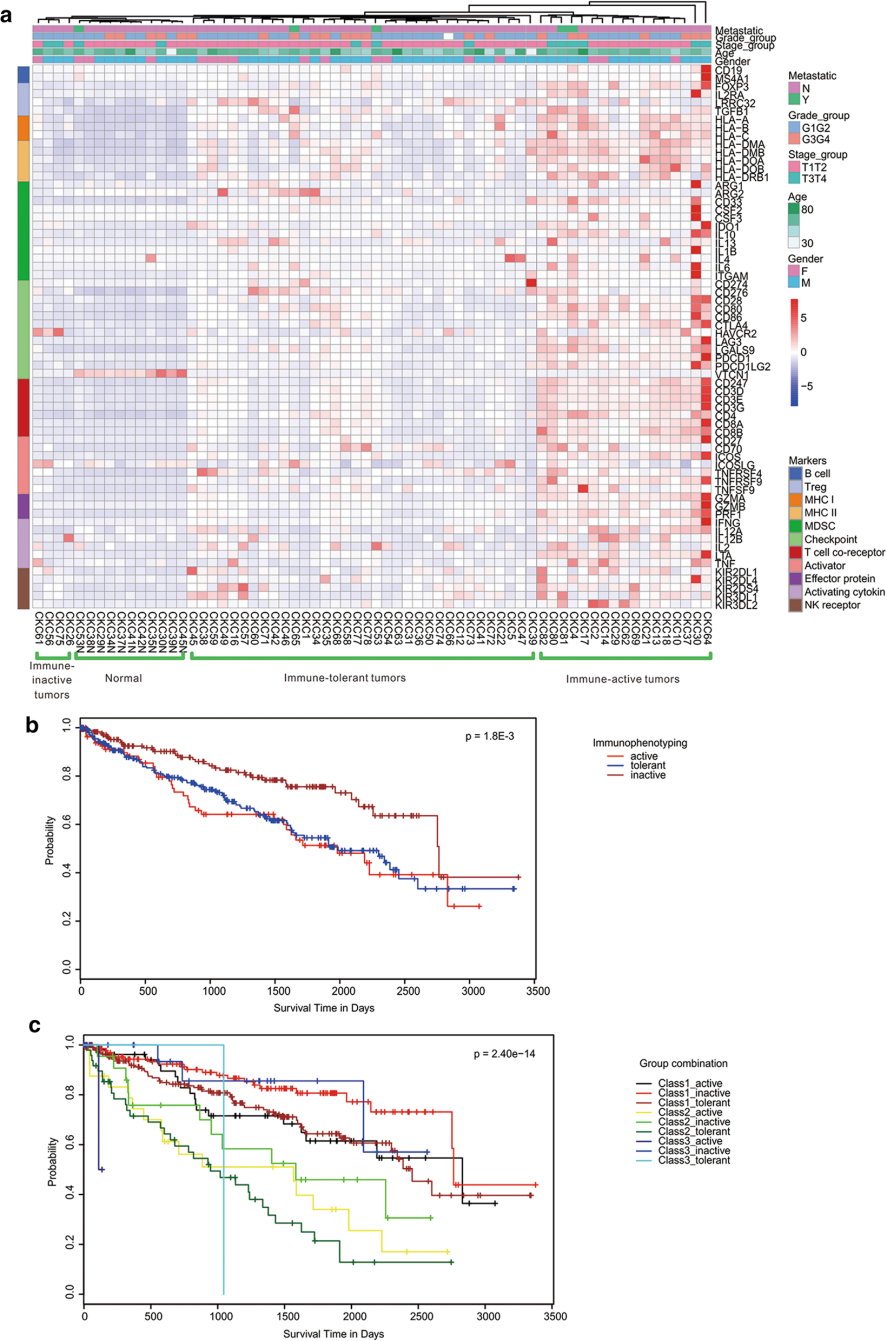
Transcriptional Characterization of Immune Microenvironment of ccRCC. **a** Unsupervised hierarchical clustering of immune gene expression within CccRCC patients (including tumor and normal samples, n = 66). A signature of 66 immune related cell markers proposed by TCGA was used for clustering. **b** Survival analysis on TCGA ccRCC patients (n = 533) grouped by three identified immuno-phenotypes (immune-active, tolerant and inactive) revealed significant difference. The log-rank test *p*-value across groups is 1.8E−3. **c** Survival analysis on combination of classification. TCGA patients were grouped by combination of molecular classification from Fig. 3 (Class 1–3) and immuno-phenotyping (immune-active, tolerant and inactive). The log rank test *p*-value across groups is 2.40E−14

To characterize tumor microenvironment in the CccRCC cohort, we first performed unsupervised hierarchical clustering of gene expression on 55 tumor and 11 matched normal samples by using a list of 66 immune markers proposed by the TCGA project [40]. Three distinct groups of tumors plus one group of normal samples were determined based on immuno-phenotyping. Seventeen tumor samples were with relatively high immune marker expression, and therefore, were defined as immune-active tumors; 4 tumor samples were clustered with normal tissues and had suppressed expression of the immune markers, hence, deemed as immune-inactive tumors; the remaining 34 tumor samples had intermediate levels of immune activity and were defined as immune-tolerant tumors (Fig. 4a). Such immune-phenotyping classification showed no observable correlation to the ccRCC classification determined in Fig. 3, suggesting immuno-phenotyping is independent of expression-based ccRCC subtypes.

When such immune-phenotyping classification was applied to the TCGA ccRCC patients, we observed significantly longer survival in the immune-inactive patients than in the immune-active and immune-tolerant patients (log-rank test *p*-value = 1.8E−3) (Fig. 4b). We further classified TCGA ccRCC patients into 9 groups using both the expression-based and immuno-phenotyping classifications, and observed more distinct survival difference than either single classification (log-rank test *p*-value = 2.40E−14), and as expected, we observed that the immune-active and immune-tolerant patients in Class 2 had the worst survival (Fig. 4c).

To identify infiltrating immune components within these samples, we performed tumor-immune interaction estimation analysis on CccRCC tumor and normal samples by the EPIC algorithm [41] (Additional file 9: Figure S5a). The composition of infiltrating immune repertoire presented remarkable heterogeneity across CccRCC patients. By comparing the immune and stroma contents in immune-active and tolerant tumors with immune-inactive tumors and normal tissues, macrophages, endothelial cells and cancer associated fibroblasts (CAFs) were observed as significantly elevated cell types in the former group (Additional file 9: Figure S5b) whereas *CD4* positive T cells are depleted, indicating macrophages and inflammation plays important role in these tumors. Moreover, as the tumor mutational burden (TMB) is reported to be associated with immunotherapy in diverse cancers [43, 44], we roughly estimated TMB (as eTMB) by the number of somatic mutations for the 11 paired samples using the RNA-seq data (Additional file 10: Table S4a). From this small sample size estimation, we did not observe clear correlation between the computed eTMB and the immunophenotyping we identified in Fig. 4a, however, we could see some evidence of the correlation between TMB and CD8+ T cells and macrophages contents in TME (Additional file 10: Table S4b).

## Discussion

This study aimed to understand the transcriptomics of Chinese ccRCC from the RNA-seq data. We first investigated gene mutations. Due to lack of sufficient matched tumor adjacent normal tissue samples and comparatively lower sensitivity of WTS-based detection, heuristic methods were used to detect and infer somatic mutations. We observed slight difference in occurrence and frequency for the previously found significantly mutated genes in TCGA ccRCC cohorts [42], except for *PBRM1*, whose mutation frequency in our cohort is much lower than in Caucasians. The RNA sequencing quality and depth at PBRM1 coding region were sufficient (Additional file 11: Table S5). Inference of gene mutation from transcriptomic data may be affected by several factors including DNA mutations that impact RNA transcript stability (e.g. through nonsense mediated decay), RNA editing that plays a role in cancer development [45], low expression of RNA that leads to undetected mutations. To evaluate whether DNA mutations affect RNA transcript stability and subsequently mRNA expression, we compared *PBRM1* expression between missense mutation, truncating mutation (some leading to nonsense mediated decay), and wild type in the TCGA clear cell renal carcinoma cohort (Additional file 12: Figure S6a). Indeed, we observed a lower *PBRM1* expression in patients with truncating mutations. However, all patients have significant *PBRM1* expression. Similarly, *PBRM1* has quite measurable expression levels in the CccRCC cohort, and no significant difference is detected between tumor and normal samples (Additional file 12: Figure S6b). Therefore, it is unlikely that DNA mutations cause low PBRM1 mutation frequency in the CccRCC cohort. From Additional file 12: Figure S6a we also observed that about 80% of *PBRM1* mutations are truncating mutations. In the 8 validated *PBRM1* mutations in the Chinese cohort, 6 or 75% are truncation mutations (Additional file 2: Table S1b). We therefore conclude that the low *PBRM1* mutation frequency in the CccRCC cohort is unlikely artifact.

*PBRM1* was previously reported to be associated with slightly worse stage and grade tumors by immunohistochemistry [46], however, the domination of early stage (78.4%) and low grade (66.1%) samples in our dataset might bias the observation. Nevertheless, remarkably low frequency of *PBRM1* mutation frequency (11%) was also reported in Polish population with a samples size of 83 patients and no bias in early stage and low grade [47]. *PBRM1* is involved in the regulation of genes of metabolic pathways that is known to be essential for driving ccRCC, including the hypoxia response related *PI3K* signaling pathway [48], high percentage of wild type *PBRM1* could be one reason why better treatment outcome of *PI3K* inhibitor was observed in the Chinese population (data not shown). *PBRM1* mutation status could also be related to immunotherapy efficacy. A recent study reported that ccRCC patients with *PBRM1* mutational inactivation benefits more from PD-1 inhibitors than those with wild type *PBRM1* [49].

We used expression data to classify ccRCC patients. Large-scale molecular diagnosis of cancer plays more and more important role in precision medicine [50, 51]. Unsupervised clustering algorithms were usually used for classifying a cancer into subtypes. Such classifications are sensitive to various parameters used in the numeric operations, such as number of genes used. From a practical and clinical point of view, a good classification should generate subtypes with distinct clinical prognosis and unique pathway activations that can be treated accordingly. As the Chinese cohort in this study lacks clinical survival information (all patients involved in this study were fortunately all alive at the time of this report), we used the TCGA ccRCC data to test our classification. The most recent multi-platform taxonomy of RCC from TCGA resulted in stratified survival groups (log rank *p*-value < 1E−7) with three ccRCC related subtypes [51], which is in favor of their previous mRNA-based clustering in 2013 [8]. Our classification further improved the stratification in overall survival. In addition to favorable distinction of survival curves, we could better correlate our classification with tumor grade levels. Although Class 1 and Class 3 tumors resulted in similar survival behavior, significant expression difference in ccRCC-driven pathways such as VEGF signaling pathway indicate that these two subtypes might be caused or impacted by distinct molecular mechanism, and should be treated differently. Using our classification, we already put more emphasis on the follow-up care of the Class 2 patients, results of which will be reported in the future.

Finally, we investigated the immuno-phenotyping of CccRCC patients. A previous study applied mass cytometry for multi-dimensional single-cell analysis on ccRCC but focused only on tumor associated macrophages and T cells due to limited number of channels [17]. Dissecting molecular signals of immune cells from TME in bulk tumor WTS by deconvolution analysis is a more convenient and cost-effective way than experimental technologies albeit resulting in insufficient resolution and precision [52–54]. Recent immunogenomic analysis on TCGA dataset identified that ccRCC is dominant of inflammatory subtype [55], which is also confirmed in CccRCC patients by our computing of cell fractions of TME cell types using the EPIC algorithm [41]. We further demonstrated that macrophages play more role in “immune-active” and “immune-tolerant” ccRCCs by comparing across samples. Further understanding of the tumor-associated macrophages might be helpful to perform respective cell-mediated immunotherapy for these patients in the future. The combination of immune-phenotyping and mRNA expression data for classifying ccRCC into 9 subtypes also gave better resolution on patient prognosis.

## Conclusion

In summary, results presented in this study shed light into the prognostic difference across populations, and gave practical guidelines on clinical treatment of ccRCC patients.

## Supplementary information

**Additional file 1.** PBRM1 primer sequences used in RT-PCR and Sanger sequencing.

**Additional file 2: Table S1. a:** this table gives the detailed information for detected driver mutation in CccRCC dataset. **b:** this table gives RT-PCR and Sanger sequencing validation of computational predicted *PBRM1* somatic mutations from RNA-Seq technology. Confirmed somatic mutation are highlighted in yellow.

**Additional file 3: Table S2.** This table contains detailed information on detected gene fusion events discovered in 55 CccRCC tumor samples.

**Additional file 4: Figure S1.** Identification of pathway variation on different clinical groups. Heatmap for gene set variation analysis (GSVA) on early (T1T2) and late (T3T4) clinical stages. Cutoffs used for GSVA were: unadjusted *p* − value < 0.01.

**Additional file 5: Figure S2.**Principle Component Analysis (PCA) on whole transcriptomes of 55 CccRCC reveals weak association between tumor stage gene expression. **a**: PCA plot colored by grade groups; **b:** PCA plot colored by stage groups.

**Additional file 6: Figure S3.** Non-negative matrix factorization (NMF) clustering for CccRCC. Heatmap for NMF classification within all CccRCC samples for ranks 2-8 using 50 iterations.

**Additional file 7: Figure S4.**Identification of gene expression based ccRCC classification. **a.** Heatmap for alignment of NMF based CccRCC classification to TCGA samples (n = 533) using 300 differentially expressed genes. **b.** Overlap of final predicted classes with original NMF-derived classes on CccRCC patients (n = 55).

**Additional file 8: Table S3.** **a**: this table gives the top 300 differentially expressed genes between three classes defined by NMF clustering algorithm. **b**: this table contains top 20 significantly enriched Reactome pathways in the top 300 differentially expressed genes between three classes defined by NMF clustering algorithm. **c**: this table summarizes the molecular classification of ccRCC samples in both Chinese and TCGA datasets.

**Additional file 9: Figure S5**. Identification of infiltrating immune cells in CccRCC. **a**. Relative fractions of tumor associated immune and stromal cells within all CccRCC samples. Samples were ordered as in the same clustering in Fig. 4. CAFs: cancer associated fibroblasts. **b**. Log10-transformed mean ratio (*x*-axis) versus *p*-value from student *t*-test for immune-active and tolerant tumors versus immune-inactive tumor and normal samples are shown. Only cell types with significant variance (*p* < 0.05) are labeled and highlighted in red (elevated) or blue (depleted).

**Additional file 10: Table S4. a**: this table gives the estimated tumor mutational burden (eTMB, the number of somatic mutations) and cell fractions from bulk expression matrix for 11 paired CccRCC samples. **b**: this table summarizes the pairwise correlation between eTMB and the corresponding cell fraction values from **a**.

**Additional file 11: Table S5. a:** this table provides whole transcriptome sequencing quality of 65 test samples in Chinese ccRCC dataset; **b:** this table summarizes the average depth of reads mapped to PBRM1 CDS region for Chinese ccRCC samples.

**Additional file 12: Figure S6.** PBRM1 expression in CccRCC and TCGA cohorts. **a** PBRM1 expression represented by log10 RSEM values are compared between missense mutation, truncating mutation and wild type in the TCGA cohort. Kruskal–Wallis test was performed across mutation type. **b** PBRM1 expression represented by log10 RSEM values are compared between tumor and normal samples in CccRCC cohort. Wilcoxon test was performed between tumor and normal.

## Data Availability

The Chinese ccRCC RNA-Seq dataset for this study is available at Gene Expression Omnibus (GEO) with accession number: GSE126964. The R scripts used for transcriptomic data analysis is available at Github repository: https://github.com/lindashirley99/CccRCC.
