# Supplementary material for: Transcriptomic characterization and innovative molecular classification of clear cell renal cell carcinoma in the Chinese population
Source: Cancer Cell Int. 2020 Sep 22;20:461. doi: 10.1186/s12935-020-01552-w (PMC7510315; doi:10.1186/s12935-020-01552-w)
Supplement: Supplementary file 1 — Additional file 1. PBRM1 primer sequences used in RT-PCR and Sanger sequencing. [file 12935_2020_1552_MOESM1_ESM.docx]

**Additional file 1**

**PBRM1 primer sequences used in RT-PCR and Sanger sequencing**

PBRM1 2616_2617------2761---2725

290 bp

PBRM1-E18-F: TGGATACTCTTGATGCTGTTATAGG

PBRM1-E18-R: ACACCTAGAAGACAGTGCATTT

atggatactcttgatgctgttatagGACAGATTCAGAAATATATGAAGATGCAGTAGAACTTCAGCAGTTTTT***TA***TTAAAATTCGTGATGAACTCTGCAAAAATGGAGAGATTCTTCTTTCACCGGCACTCAGCTATACCACAAAACATTTGCATAATGATGTGGAGAAAGAGAGAAAGG***A***AAAATTGCCAAAAGAAATAGAGGAAGATAAACTAAAA***C***GAGAAGAAGAAAAAAGAGgttggtttcttttcattttattgaatatgaaaaaatgcactgtcttctaggtgttctcataattagctgtggaaaagagtcctgtttgtatgaatgaaaactcagggtattgactatattagagtagacg

PBRM1 2125_2126------2492

490 bp

PBRM1-E17-F: TCTGAGTTGCCTGACTACTATCT

PBRM1-E17-R: TCCTTCTTGCTCGTTCCAATAC

GTAGGAAGAGTGGCATTTCTCCTAAAAAATCAAAATACATGACTCCAATGCAGCAGAAACTAAATGAGGTCTATGAAGCTGTAAAGAACTATACTGATAAGAGGGGTCGCCGCCTCAGTGCCATATTTCTGAGGCTTCCCTCTAGATCTGAGTTGCCTGACTACTATCTGACTATTAAAAAGCCCATGGACATGGAAAAA***AT***TCGAAGTCACATGATGGCCAACAAGTACCAAGATATTGACTCTATGGTTGAGGACTTTGTCATGATGTTTAATAATGCCTGTACATACAATGAGCCGGAGTCTTTGATCTACAAAGATGCTCTTGTTCTACACAAAGTCCTGCTTGAAACACGCAGAGACCTGGAGGGAGATGAGGACTCTCATGTCCCAAATGTGACTTTGCTGATTCAAGAGCTTATCCACAATCTTTTTGTGTCAGTCATGAGTCATCAGGATGATGAGGGAAGATGCTACAGCGATTCTTTAGCAGAAATTCCTGCTGTGGATCCCAACTTTCCTAACAAACCACCCCTTACATTTGACATAATTAGGAAGAATGTTGAAA***A***TAATCGCTACCGTCGGCTTGATTTATTTCAAGAGCATATGTTTGAAGTATTGGAACGAGCAAGAAGGATGAATCGgtatgttttcaaagccatttttatt

PBRM1 58_59

158 bp

PBRM1-E2-F: AAGAAGTTGGATTCCATGGGT

PBRM1-E2-R: CTCACAGGATCTACAGTTGGAAG

catggctgattatttcttatagaagAAGTTGGATTCCATGGGTTCCAAGAGAAGAAGAGCTACCTCCCCTTCCAGCAGTGTCAGCGGGGACTTT***GA***TGATGGGCACCATTCTGTGTCAACACCAGGCCCAAGCAGGAAAAGGAGGAGACTTTCCAATCTTCCAACTGTAGATCCTgtgagtaacttggattacatggttt

PBRM1 3177

209 bp

PBRM1-E21-F: AACTTCCGAGATGAGGATGTTT

PBRM1-E21-R: CTGTCCTCTGAGTTGTCTGTATTC

GAATACTTTAAGTTATGCCCAGAAAACTTCCGAGATGAGGATGTTTTTGTCTGTGAATCACGGTATTCTGCCAAAACCAAATCTTTTAAGAAAATTAAACTGTGGACCATGCCCATCAGCTCAGTCAG***G***TTTGTCCCTCGGGATGTGCCTCTGCCTGTGGTTCGCGTGGCCTCTGTATTTGCAAATGCAGATAAAGGTGATGATGAGAAGAATACAGACAACTCAGAGGACAGTCGAGCTGAAGACAATTTTAACTTGGAAAAG

PBRM1 4391

233 bp

PBRM1-E29-F: ATGTGGTCCTGTGCTCTTTC

PBRM1-E29-R: GTGTCTGATCCCACTTGCTAAT

accatgtggtcctgtgctctttcagGTGGGAGTTTTGGGGCCTCCAGGGCAGCAGG***C***ACCACCTCCATATCCCGGCCCACATCCAGCTGGACCCCCTGTCATACAGCAGCCAACAACACCCATGTTTGTAGCTCCCCCACCAAAGACCCAGCGGCTTCTTCACTCAGAGGCCTACCTGAAATACATTGAAGGACTCAGTGCGGAGTCCAACAGCATTAGCAAGTGGGATCAGACACTGGCAG

PBRM1 3296

315 bp

PBRM1-3296-F: AAATTAAACTGTGGACCATGCC

PBRM1-3296-R: TCTGTTATAGTTTCCTTCTTCCTGT

GAATACTTTAAGTTATGCCCAGAAAACTTCCGAGATGAGGATGTTTTTGTCTGTGAATCACGGTATTCTGCCAAAACCAAATCTTTTAAGAAAATTAAACTGTGGACCATGCCCATCAGCTCAGTCAGGTTTGTCCCTCGGGATGTGCCTCTGCCTGTGGTTCGCGTGGCCTCTGTATTTGCAAATGCAGATAAAGGTGATGATGAGAAGAATACAGACAACTCAGAGGACAGTCGAGCTGAAGAC***AAT***TTTAACTTGGAAAAGgtatgcagataatagccacacagaaaaaattttacatctcaaaacacctattccagaaaataaatgaataaagtattcaaacttagaaaaagagcaacaacacaaaaccttagccaaacaggaagaaggaaactataacagaaagaaatttacactttcaaaactgtacattataatttaaaag

PBRM1 3784

320 bp

PBRM1-3784-F: AGGCCAACTGAAATACCAGAAA

PBRM1-3784-R: CAGGTCCCAGCAGATTATGAAG

GAAAGTGTGCTGTGTTGTCATTCAAGGACTTCCTCTCCTGCAGGCCAACTGAAATACCAGAAAATGACATTCTGCTTTGTGAGAGCCGCTACAATGAGAGCGACAAGCAGATGAAGAAATTCAAAGGATTGAAGAGGTTTTCACTCTCTGCTAAAGTGGTAGATGAT***GAA***ATTTACTACTTCAGgtaaagcttgaaaaacttaaggaaaaaagagcacttccattaactgatagcaacatagtgtagtccagtgtttttaattttttattagatcttaaactagagtagtaaatatttcaaatagaaaatatttattcacttaaagattaaaggaaagacttcataatctgctgggacctggtggctgctctaatagtgcccttggcgtagactccaataattgtggaatgaggcagaaacacttggttctggtttatatcctgtcaggagatttctcagttc

PBRM1 335

240 bp

PBRM1-335-F: CCAGACTATTATGAAGTGGTTTCTC

PBRM1-335-R: GAAACTACTACTCACCTGCCA

tttaaaaaaatttaaattataacagAAATCAACCAGACTATTATGAAGTGGTTTCTCAGCCCATTGACTTGATGAAAATCCAACAGAAACTAAAAATGGAAGAGTATGATGATGTTAATTTG***CTG***ACTGCTGACTTCCAGCTTCTTTTTAACAATGCAAAGTCCTATTATAAGgtaagaaattatgaaatttggaaagataccaattggaagataccaatttgataattggcttcttaatatttataaacctggcaggtgagtagtagtttctttttgtggcttgagttcgcatttgtcttattcgtaaagagactggac

PBRM1 4286

251 bp

PBRM1-4286-F: CCCAGTTGATGGCCTTGTTA

PBRM1-4286-R: TGCTGACAACCTTTGGATTCT

GCATGATGGGTGGCTATCCGCCAGGCCTTCCACCTTTGCAGGGCCCAGTTGATGGCCTTGTTAGCATGGGCAGCATGCAGCCACTTCACCCTGGGGGGCCTCCACCCCACCATCTTCCGCCAGGTGTGCCTGGCCTCCCGGGCATCCCACCA***CCG***Ggtaagaacttcatcctcattcactcattaatctcatcttcatattctctttttccgctttcaaccagttgttccaggaggcacccgtggcccattgtggccaggccttccctccctgagaatccaaaggttgtcagcagcagggcatgtcttgtgcattcagcaggtggcccagcacttgtgcccttgtgcac

PBRM1 chr3:52584654 Allele: A

511 bp

PBRM1-52584654-F: GATTCAAGGGCAGAGGTACATAG

PBRM1-52584654-R: GTGTCTGATCCCACTTGCTAAT

tacctagagaaatactagaattttatgcaaggttcaaatcatttctaagccataattgatgtacatctgacagattgttctagtattatactttaagtacctagaaacaaaacaactttctattcaaatcctattggggtgcttaagattcctagccacaaagattcaagggcagaggtacataggtttgaattatgaatggcatcatttcttatcagacagtagtttttcagtcatttcaacccagagcctctaactgtgccattgcattctgaattataggtgtgatgaaccaaggagtggcccctatggtagggactccagcaccaggtggaagtccatatggacaacaggtgagcctcccagtttgattttctaggacttgacagaattcgagttatccttctcagaacatgtgcagagtctctttttgcctcaccatgtggtcctgtgctctttca***g***GTGGGAGTTTTGGGGCCTCCAGGGCAGCAGGCACCACCTCCATATCCCGGCCCACATCCAGCTGGACCCCCTGTCATACAGCAGCCAACAACACCCATGTTTGTAGCTCCCCCACCAAAGACCCAGCGGCTTCTTCACTCAGAGGCCTACCTGAAATACATTGAAGGACTCAGTGCGGAGTCCAACAGCATTAGCAAGTGGGATCAGACACTGGCAG
