# Supplementary figures and images for: Transcriptomic characterization and innovative molecular classification of clear cell renal cell carcinoma in the Chinese population
Source: Cancer Cell Int. 2020 Sep 22;20:461. doi: 10.1186/s12935-020-01552-w (PMC7510315; doi:10.1186/s12935-020-01552-w)

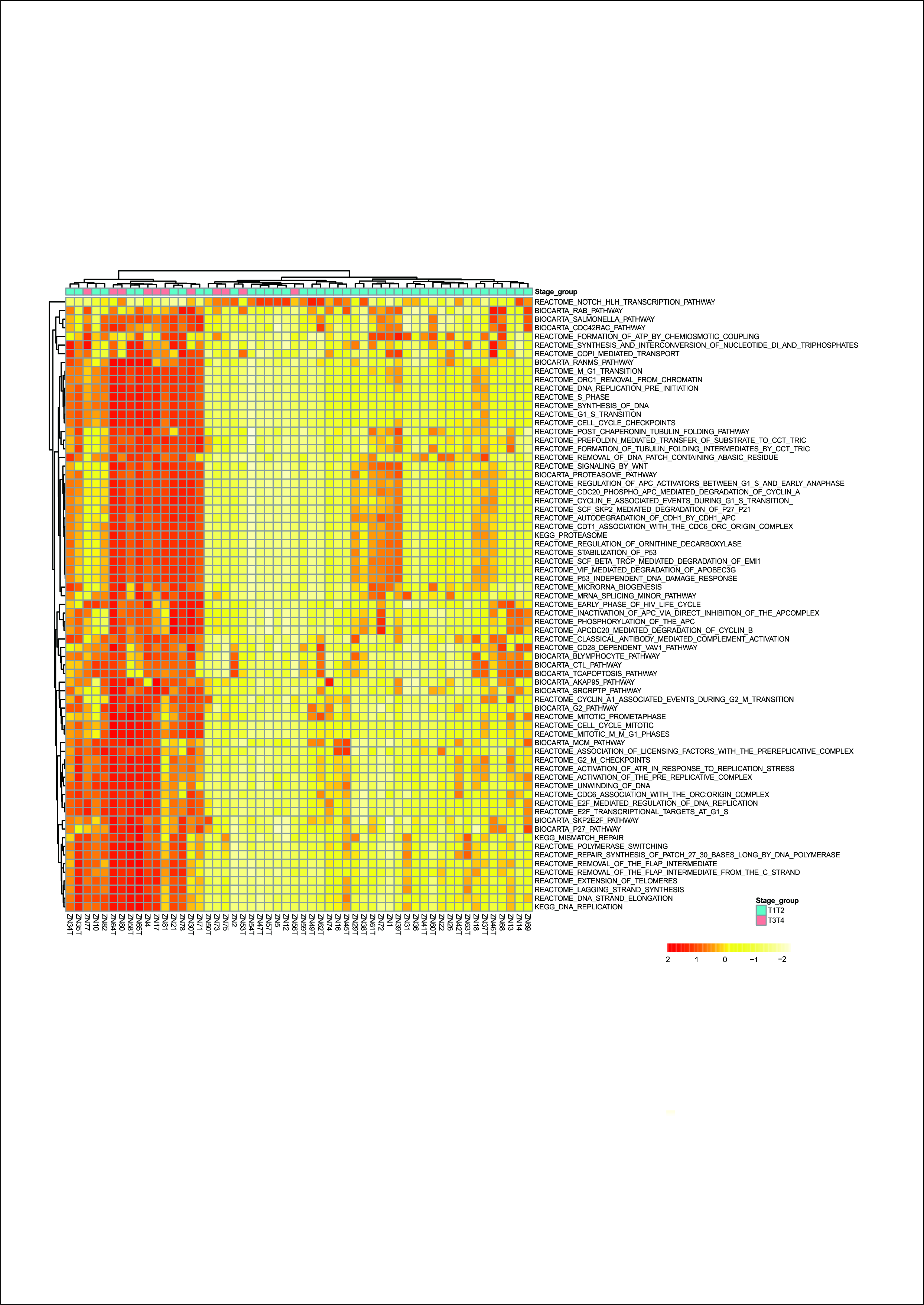

Supplement: Supplementary file 4 — Additional file 4: Figure S1. Identification of pathway variation on different clinical groups. Heatmap for gene set variation analysis (GSVA) on early (T1T2) and late (T3T4) clinical stages. Cutoffs used for GSVA were: unadjusted p − value < 0.01. [file 12935_2020_1552_MOESM4_ESM.tif]

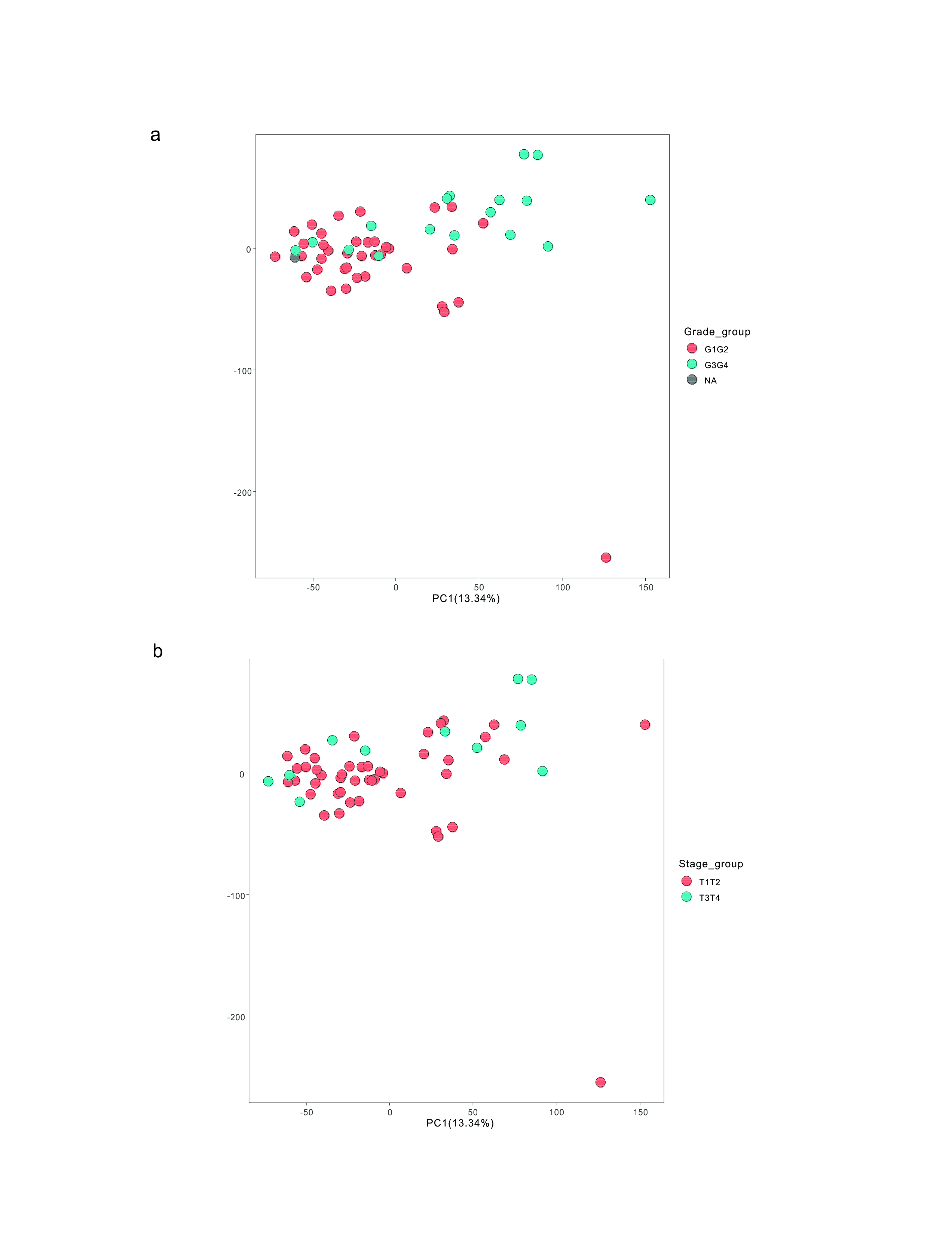

Supplement: Supplementary file 5 — Additional file 5: Figure S2.Principle Component Analysis (PCA) on whole transcriptomes of 55 CccRCC reveals weak association between tumor stage gene expression. a: PCA plot colored by grade groups; b: PCA plot colored by stage groups. [file 12935_2020_1552_MOESM5_ESM.tif]

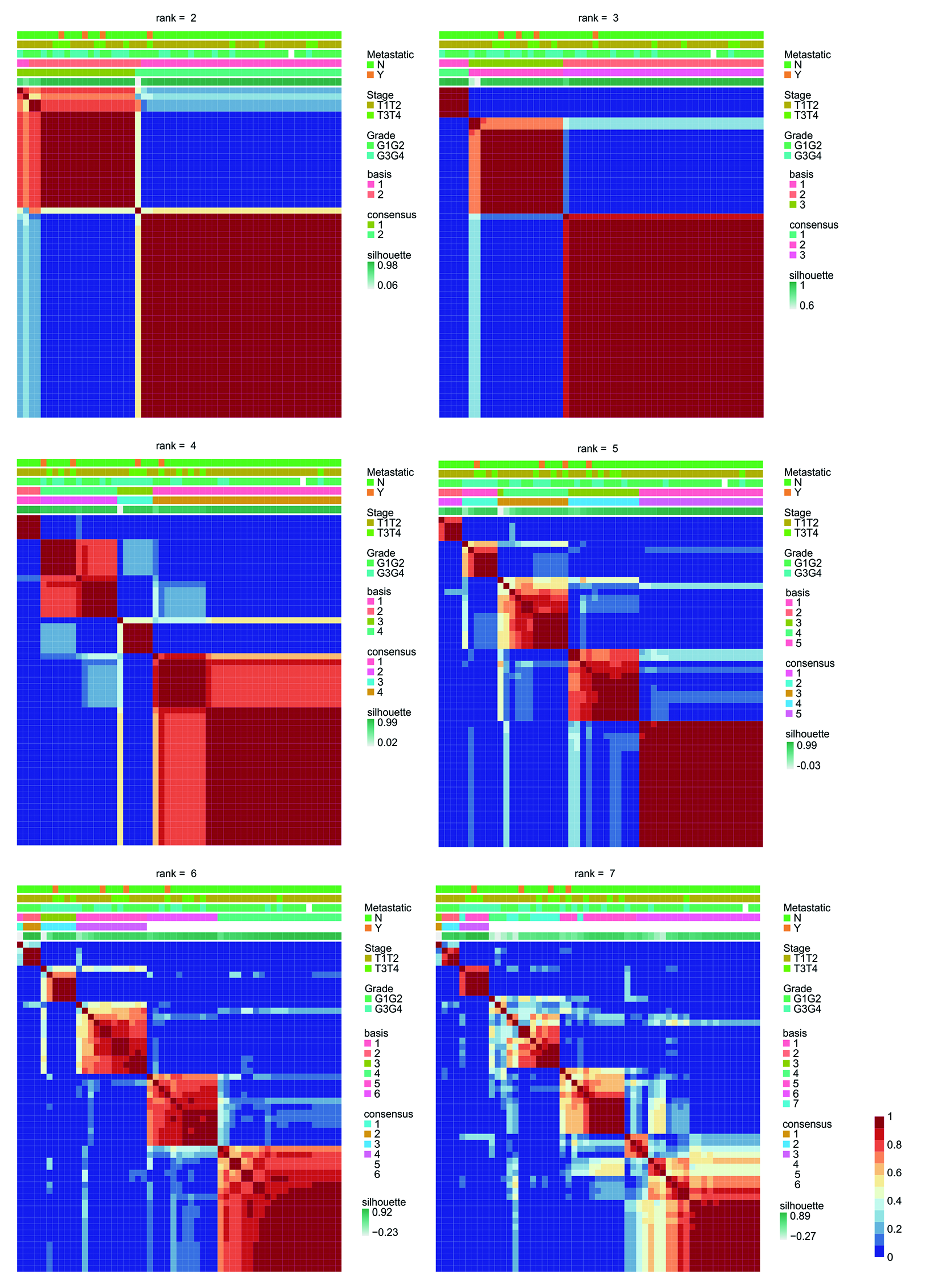

Supplement: Supplementary file 6 — Additional file 6: Figure S3. Non-negative matrix factorization (NMF) clustering for CccRCC. Heatmap for NMF classification within all CccRCC samples for ranks 2-8 using 50 iterations. [file 12935_2020_1552_MOESM6_ESM.tif]

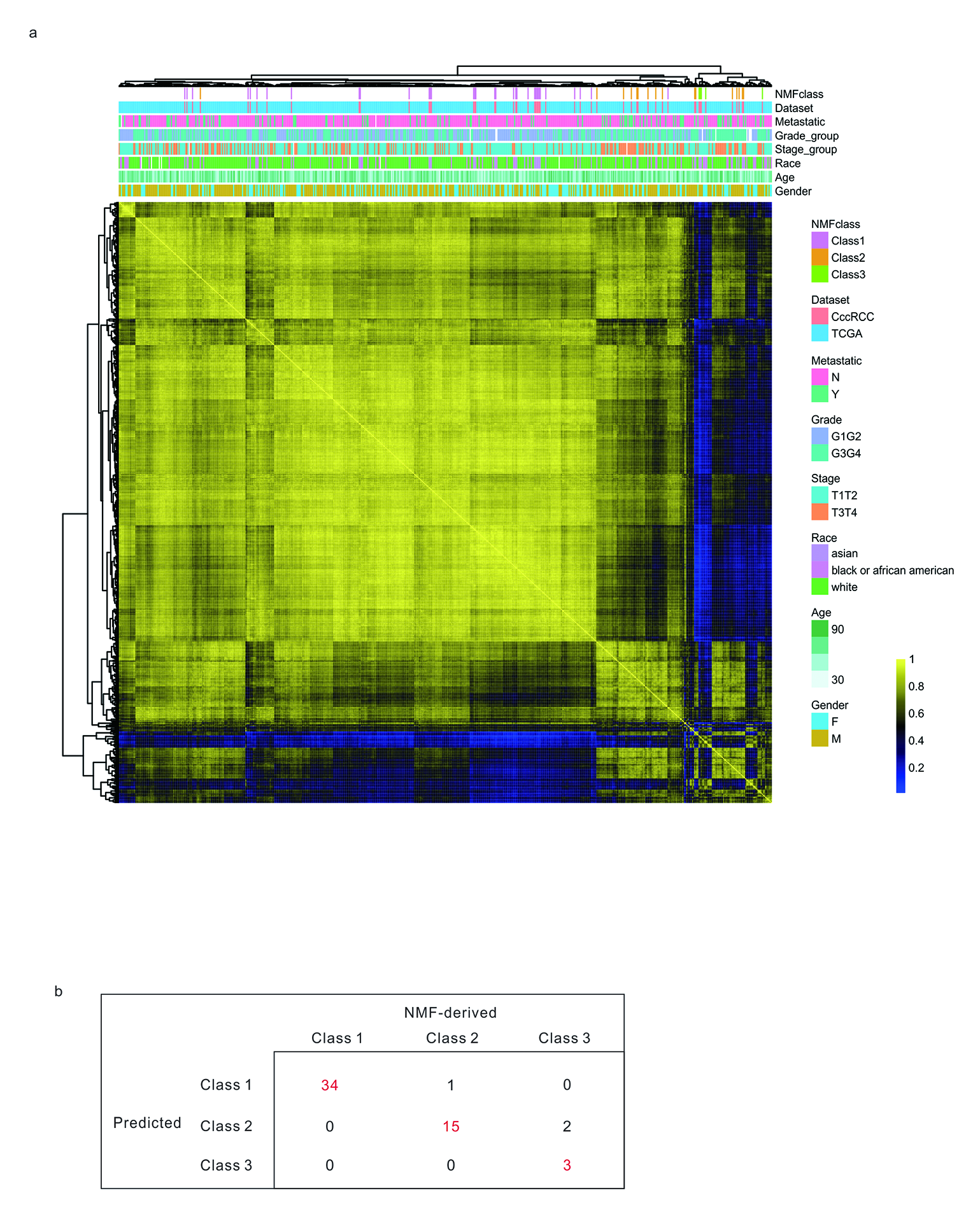

Supplement: Supplementary file 7 — Additional file 7: Figure S4.Identification of gene expression based ccRCC classification. a. Heatmap for alignment of NMF based CccRCC classification to TCGA samples (n = 533) using 300 differentially expressed genes. b. Overlap of final predicted classes with original NMF-derived classes on CccRCC patients (n = 55). [file 12935_2020_1552_MOESM7_ESM.tif]

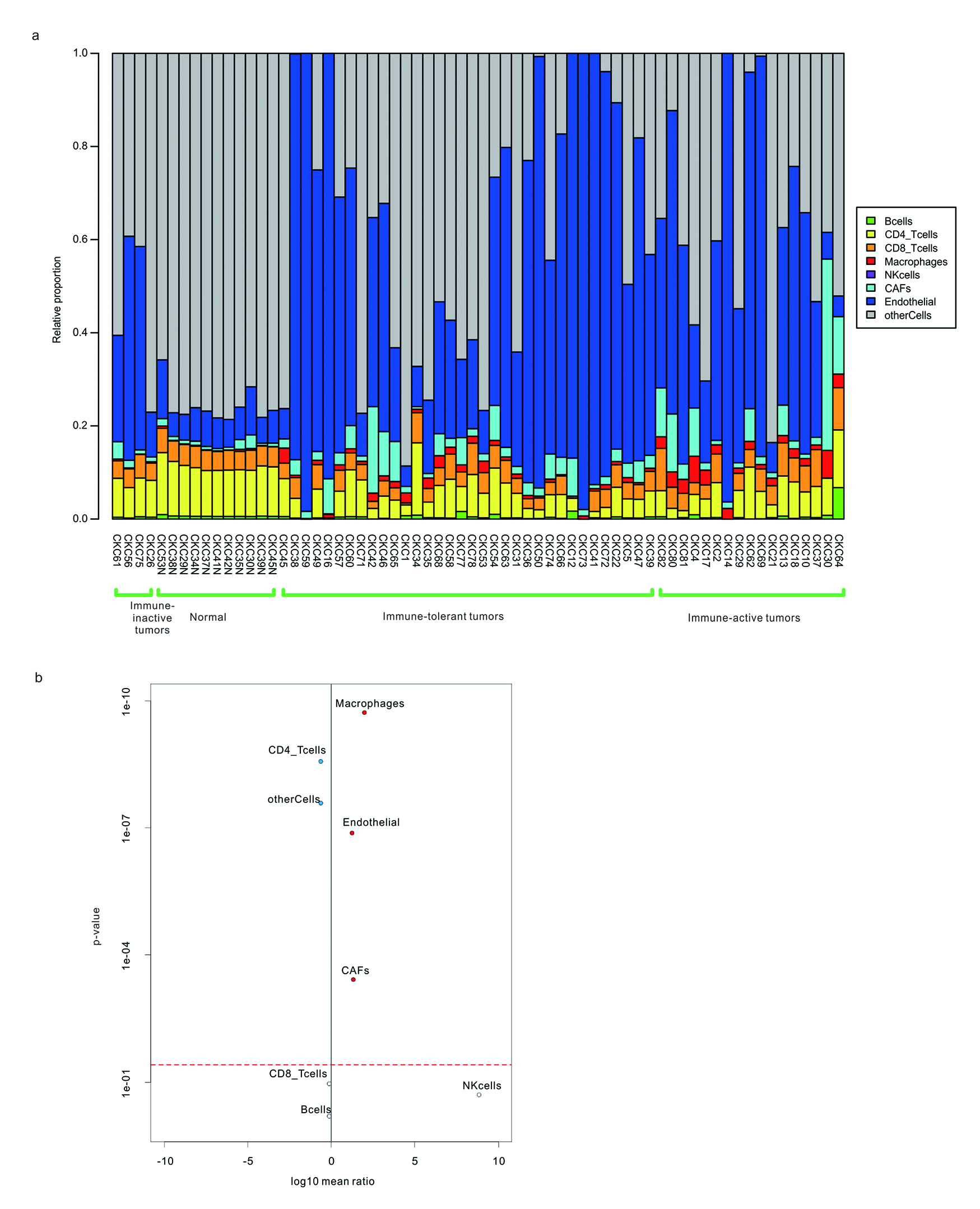

Supplement: Supplementary file 9 — Additional file 9: Figure S5. Identification of infiltrating immune cells in CccRCC. a. Relative fractions of tumor associated immune and stromal cells within all CccRCC samples. Samples were ordered as in the same clustering in Fig. 4. CAFs: cancer associated fibroblasts. b. Log10-transformed mean ratio (x-axis) versus p-value from student t-test for immune-active and tolerant tumors versus immune-inactive tumor and normal samples are shown. Only cell types with significant variance (p < 0.05) are labeled and highlighted in red (elevated) or blue (depleted). [file 12935_2020_1552_MOESM9_ESM.tif]

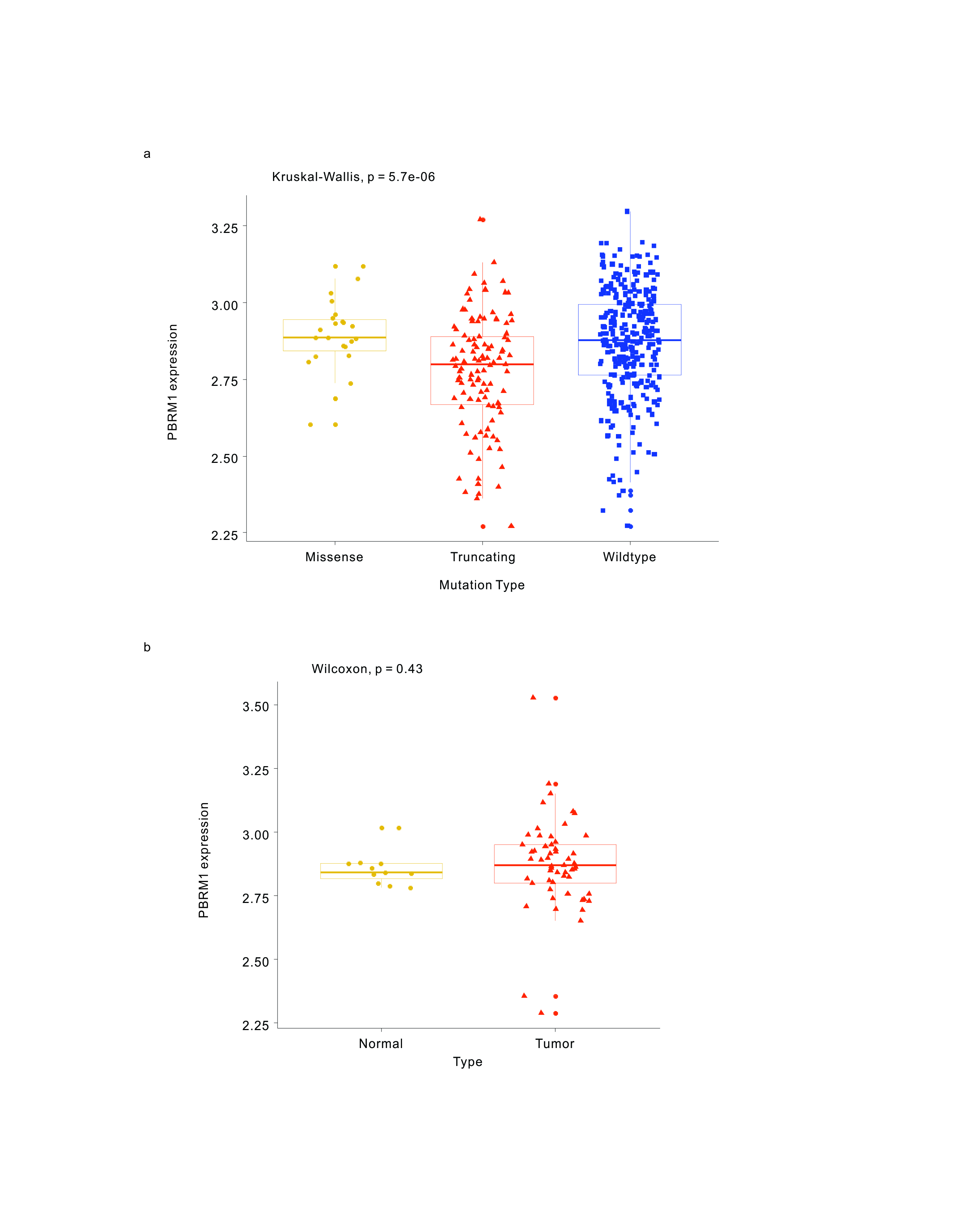

Supplement: Supplementary file 12 — Additional file 12: Figure S6. PBRM1 expression in CccRCC and TCGA cohorts. a PBRM1 expression represented by log10 RSEM values are compared between missense mutation, truncating mutation and wild type in the TCGA cohort. Kruskal–Wallis test was performed across mutation type. b PBRM1 expression represented by log10 RSEM values are compared between tumor and normal samples in CccRCC cohort. Wilcoxon test was performed between tumor and normal. [file 12935_2020_1552_MOESM12_ESM.tif]
